# Trajectories of Procedural and Interactional Justice as Predictors of Retirement among Swedish Workers: Differences between Three Groups of Retirees

**DOI:** 10.3390/ijerph18126472

**Published:** 2021-06-15

**Authors:** Constanze Eib, Paraskevi Peristera, Claudia Bernhard-Oettel, Constanze Leineweber

**Affiliations:** 1 Department of Psychology, Uppsala University, 752 37 Uppsala, Sweden; 2 Stress Research Institute, Stockholm University, 114 19 Stockholm, Sweden; paraskevi.peristera@su.se (P.P.); constanze.leineweber@su.se (C.L.); 3 Department of Psychology, Stockholm University, 114 19 Stockholm, Sweden; cbl@psychology.su.se

**Keywords:** organizational justice, fairness, retirement, trajectories, self-rated health, Sweden

## Abstract

Organizational justice is an important aspect of the psychosocial work environment, but there is a lack of studies on whether justice perceptions also predict retirement decisions. The aim of this study is to examine trajectories of procedural and interactional justice perceptions prior to retirement of three groups of retirees while considering self-rated health and important demographics. Data from the Swedish Longitudinal Occupational Survey of Health (2006–2018, N = 3000) were used. Respondents were grouped into early retirement, normative retirement and late retirement. Latent growth curve models and multinomial logistic regressions were conducted to test whether trajectories of justice perceptions prior to retirement differed between retirement groups while controlling for self-rated health development and demographic variables. Late retirees had higher intercept levels of interactional justice and higher intercept levels of self-rated health prior to retirement, compared to early retirees. Late retirees also showed a slower decrease in procedural justice compared to early retirees. Only intercept levels of self-rated health differed between early retirees and normative retirees, such that early retirees had lower levels of self-rated health prior to retirement. Keeping employees in the workforce is a major challenge for any aging society. Organizational justice perceptions in the years prior to retirement seem particularly influential for delaying retirement.

## 1. Introduction

In Europe, decreasing birth rates together with longer time spent in higher education and a later entry into the labor market make it vital for organizations to rely more on older workers. The European Union (EU) projects that while around 19% of all people in the EU currently are aged 65 or older, in 2080 that percentage will be 29% [1]; that is close to one out of three inhabitants. Traditionally, in many European countries the age of 65 has been seen as the “normal” retirement age and this view is still persistent [2], although policies that extend working lives have been implemented in several countries. In Sweden, there is officially no statutory retirement age. However, the norm is to leave working life at the age of 65 years [3,4]. Until recently, workers in Sweden had the opportunity to retire from the age of 61 and receive earnings-related state pension. From the age of 65 years, a guarantee pension is paid out to all if the earnings-related state pension is below a certain threshold. Many workers also have an occupational pension from their employers, this type of pension is usually paid out at the age of 65 years although individuals can actively change the timing of payments. In the following, we refer to individuals retiring around 65 years as “normative retirement” to highlight the socially constructed norm. Hence, many organizations face the question how to influence individuals’ retirement behavior and retirement decision-making processes.

Psychosocial work factors have been shown to shorten, as well as extend working lives [5,6], but also health is an important factor for retirement decisions [7,8,9]. A recent systematic review on the relationship between psychosocial environment and actual retirement found that high job control had positive effects on retiring late, job demands showed inconsistent effects, while the number of studies about the effects of job insecurity and effort-reward imbalance was insufficient to draw firm conclusions [10]. Moreover, few studies investigated early retirement (excluding disability pension), but some indicated that high demands may be associated with early retirement intentions [10]. Apart from these factors, an important psychosocial work factor is organizational justice; the subjective evaluation of the employer’s fairness [11]. While the early justice literature has focused on distributive justice (the perceived fairness of outcome allocations), procedural justice (the perceived fairness of the procedures and processes that lead to outcome allocations) has received the most research attention as predictor of employees’ work and health outcomes [12]. In addition to these two facets, researchers have established that interpersonal justice (the perceived fairness of interpersonal treatment often by the immediate line manager) and informational justice (the perceived fairness of explanations and justifications given) contribute to a fuller understanding of the organizational justice concept [13]. The latter two facets are sometimes combined into interactional justice. Whereas distributive and procedural justice are seen more as lying within the vicinity of the organization, interactional justice can be impacted more easily by individual line managers [14]. The present paper uses both procedural justice and interactional justice as separate predictors of retirement behavior, which allows to better disentangle whether retirement behavior is impacted more by aspects that organizations have more control over or whether retirement behavior is more impacted by aspects that individual line managers can control.

An extensive number of studies has shown that organizational justice facets have substantial relations with work and health outcomes [12,15,16,17]. Particularly procedural justice and interactional justice communicate to employees that they are valued members of the work group, that they are respected and have a high standing within the work group [18]. Social exchange theory posits that employees who feel treated fairly are more likely to reciprocate fairness, and continue working with their employer, something which has been shown to be a valid explanation in empirical studies [12,19]. In contrast, employees who experience low procedural and interactional justice have a higher likelihood of leaving the work group and employer [20,21]. One decisive way of leaving the employer is to leave the labor market altogether, that is, retiring.

Research regarding the potential influence of organizational justice perceptions on retirement decisions is scarce. Especially studies that distinguish between individuals that stopped working before normative retirement age versus those who continued working after the normative retirement age are lacking. In two prospective studies from Denmark, it was found that employees had a higher rate of early retirement (exclusive disability pension) in work units with lower levels of organizational justice even when controlling for health [22,23]. In a prospective cohort study from Finland, higher values of procedural and interactional justice were associated with a decreased risk of disability pension from all-causes, depression and musculoskeletal diseases [24]. However, significant effects disappeared after including job strain and effort-reward imbalance. There are slightly more studies on predicting intentions (instead of behavior) to retire early, however, these studies are all based on cross-sectional data and stem exclusively from Finland, which limits generalizability to other countries [25,26,27,28]. These studies found that retirement intentions were higher with lower levels of procedural and interactional justice. As retirement is a process that takes place over time [9], longitudinal data with multiple points of measurement over time before retirement are needed to investigate retirement behavior and its antecedents [8].

Apart from work characteristics, relevant antecedents for retirement decisions are manifold. The most frequently studied cause for early retirement is poor health [8]. Longitudinal studies revealed that poor self-rated health is a strong predictor of labor market exit through disability, unemployment, and early retirement [29,30]. However, none of these studies investigated health trajectories as predictor of retirement, that is, the development of self-rated health, during the years before retirement. Additionally, it is unclear whether good health actually prolongs working life or rather means that the normative retirement age can be reached. Further, the influence of poor health on early retirement due to other reasons than disability pension (e.g., voluntary retirement) is less studied. In a study including Finnish civil servants, poor mental health was associated with increased odds of subsequent voluntary early retirement [31]. Moreover, poor physical functioning was associated with increased odds of normative retirement (compared with continuing work or having left work for reasons other than retirement). In a French cohort study, self-rated ill-health increased before retirement [32], and in two Finnish cohorts, changes in self-rated health during retirement transition related mainly to occupational status, such that those with higher occupational status had more beneficial health developments than those with lower status [33]. Thus, when attempting to unravel whether organizational justice developments during the years before retirement can add to the understanding of decisions to retire it is of importance to take self-rated health developments prior to retirement into consideration. In the current study, we aim to investigate trajectories in procedural and interactional justice and self-rated health in relation to retirement decisions. More specifically, we study whether developments of procedural and interactional justice at the end of working life predict whether one retires early, at the normative age or later. In addition, developments in self-rated health prior to retirement are investigated.

## 2. Materials and Methods

### 2.1. Data and Sample

Data were drawn from the Swedish Longitudinal Occupational Survey of Health (SLOSH) study, which is a national cohort study with data being collected biennially. Data collection started in 2006 with a follow-up of participants of the Swedish Work Environment Survey (SWES) 2003, conducted by Statistics Sweden. SWES consist of a subsample of gainfully employed people aged 16–64 from the Labour Force Survey (LFS). These individuals are first sampled into LFS through stratification by county, sex, citizenship and inferred employment status. Thus, SLOSH is approximately representative of the Swedish working population. In later SLOSH waves, additional SWES cohorts were subsequently added and today SLOSH comprises SWES participants from 2003 until 2011, including a sample size of over 40,000 individuals [34].

The SLOSH study questionnaire comes in two versions; one questionnaire for the working population (for those working at least 30% or more) and one for the non-working population (for those working less than 30% or not working at all, e.g., for participants who have temporarily or permanently left working life). 

For this study, we used data collected between 2006 and 2018 (response rates between 65% (*n* = 5985) and 48% (*n* = 17,841)). We included all individuals that were equal or above 50 years old, and have given answers either to the working or the non-working questionnaire. We defined retirees as individuals that in the non-working questionnaire had one of the following answers: old-age retirement or receiving another sort of pension on a full-time basis (exclusive disability pension). We also included those who were still working after the age of 66 years. In addition, individuals who were re-employed (those who returned back to work after their first full-time retirement and answered to the working questionnaire again) were included. However, these participants only contributed to the analyses with their first work–retirement transition. Participants who left work due to other reasons (e.g., disability pension, death, etc.) were excluded from all analyses. In total, data are available for N = 3000.

### 2.2. Retirement Groups

Based on the pension system in Sweden, participants were classified into three retirement groups. Age regards age at the end of the year, thus a person here classified as being 66 might actually still have been 65 when answering the questionnaire (which is sent out during March/April). Normative retirement was defined as retiring between the 65–66 years of age (retirement group = 1, N = 1231, M_age_ = 65.23, SD_age_ = 0.42, range = 65–66). Early retirement was defined as having retired at or before the age of 64 years (early retirement = 2, N = 952, M_age_ = 62.82, SD_age_ = 1.48, range = 52–64), and late retirement included participants who retired above the age of 66 years or were still working after their 66th birthday (late retirement = 3, N = 817). In the third category, we included all participants who retired after the age of 66 years (N = 614, M_age_ = 67.73, SD_age_ = 1.32, range = 67–79). In this group, we also included those who filled out the questionnaire for those in work despite being 66 years or older (N = 401 M_age_ = 67.73, SD_age_ = 2.07, range = 66–80). 

Data were rearranged in the way that the time point T0 is the year of retirement or last wave for those who were 66 years or older and had not made a retirement transition yet. Relative to T0, we considered the previous time points, to observe organizational justice and self-rated health developments when participants were still working. To that end, we ordered time backwards, and with a two-year gap between questionnaire send-outs.

### 2.3. Measures

#### 2.3.1. Procedural Justice

Seven items concerned the fairness of decision-making processes [35]. The items were instructed with “The following statements relate to the organization’s decision-making process” and an example item is: “All sides affected by the decision are represented” (for a full item list, see Table 1). Items were answered on a 5-point Likert scale ranging from 1 = “strongly disagree” to 5 = “strongly agree”. To calculate a procedural justice measure for each wave, responses were reversed and summed up when ≥4 of the 7 items were answered. Higher values reflect more positive perceptions of procedural justice. The measure of procedural justice was included into the questionnaire for the working population in 2006. Thus, for procedural justice data from a total of seven waves were available (2006–2018).

#### 2.3.2. Interactional Justice

Was measured with seven items that have been validated in studies on organizational justice before [20,36]. An example item is “I receive praise from my boss if I have done something good” (for a full item list, see Table 1). Participants answer on a four-point Likert-scale ranging from 1 = “yes, often” to 4 = “no, never”. Responses were revised in the way that higher values indicated more interactional justice. To calculate an interactional justice measure for each wave, responses were summed up when ≥4 of the 7 items were answered. The measure of interactional justice was included into the questionnaire for the working population in 2010, and thus, data from five waves (2010–2018) were available for this paper.

#### 2.3.3. Self-Rated Health

Self-rated health was measured with a single question “How would you rate your general state of health?” answered on a five-point scale reaching from “very good” to “very bad”. Reliability and validity of this one-item self-rated health measure has been established [37]. Before analyses, responses were reversed, so that higher values indicate better self-rated health.

#### 2.3.4. Demographic Characteristics

In line with reviews on the impact of demographic variables on retirement behavior [7,9], we controlled for sex, income, socioeconomic status, and marital status; all recorded one wave before retirement (or at last wave of answering for those still in work). Sex (0 = men/1 = women) and income (gross income in Swedish thousands of crowns, in the analysis for hypothesis testing, ln(income) was used) were obtained by linkage to registry data. Socioeconomic status, based on the Swedish socioeconomic classification (SEI; 0 = blue/1 = white-collar), and marital status (0 = single/1 = married, cohabiting) were derived from questionnaire data.

### 2.4. Statistical Analyses

In the first step, latent growth curve models (LGCM) were fitted to our data [38,39]. These models are useful for tracking intra-individual changes of trajectories over time and examining predictors of individual differences in change. Since LGCMs are developed using structural equation modeling (SEM), they consider change over time in terms of an underlying, latent, unobserved process. In the case of linear trajectories, they are therefore broken down into two latent constructs, the intercept factor, which represents the level, and a slope that describes the rate of change. The variance, that the models estimate, indicates individual differences. These latent constructs of the trajectories can also be included in models as predictors to an outcome. LGCMs’ have the advantage to handle missing data using full information maximum likelihood (FIML) estimation, as well as that they can be adjusted for measurement error.

In our analyses, procedural justice included repeated measurements over four waves prior to retirement, interactional justice used three waves prior to retirement, while self-rated health was evaluated with both three and four measurements prior retirement. In a first step, we estimated four types of unconditional linear models, which specified repeated measures of the outcomes as a function of (a) fixed intercept (Model 0), (b) random intercept (Model 1), (c) random intercept and fixed slope (Model 2), and (d) random intercept and slope (Model 4) with the aim to determine which model fits best the data. In the case of procedural justice, Model 0 was a fixed intercept model, which estimates the average level of procedural justice for all individuals across time, with no variation within or between individuals across time. Model 1 was a random intercept model, which tests the variation between individuals in their level of procedural justice. Model 2 was a random intercept, fixed slope model, which estimates the average rate of change in procedural justice over time for all individuals. Finally, Model 3 was a model with random intercept and random slope, which estimates whether there is sufficient variation in the change of procedural justice over time.

All latent growth curve models (LGCMs) were obtained through FIML estimation, using MPLUS 8 [40]. Chi-square difference test, sample-sized adjusted Bayesian information criterion (SBIC) scores, comparative fit index (CFI), root mean square error of approximation (RMSEA) and standardized root mean square (SRMR) were used to assess goodness of fit of the different models. CFI scores greater than 0.95, RMSEA and SRMR less than 0.08 are indicators of good fitting [41,42]. The chi-square difference test compared Model 3 to Model 2 and Model 2 to Model 1. The smaller the Chi-square and the SBIC the better fits the model.

In a second step, we run multinomial logistic regressions with the dependent variable being the retirement group (a categorical variable with three categories) and the independent variables being the intercept and slope coefficients derived from the best model obtained from the LGCMs. The steps of the regressions were the following: Model 1 included either procedural or interactional justice, Model 2 added self-rated health (Model 1 + self-rated health), and Model 3 added demographic characteristics (Model 2 + demographic characteristics). Odds ratios and 95% confidence intervals (CI), as well as −2 log likelihood and Cox and Snell Pseudo R statistic are provided. Multicollinearity, linearity, independence of errors, as well as residuals were examined and results showed no signs of concerns [43].

## 3. Results

### 3.1. Descriptive Results for the Different Retirement Groups

Descriptive characteristics are provided in Table 2. Participants who retired at the normative retirement age were to a higher percentage blue-collar worker and had the lowest average income. Those with a late retirement age were more often men, less often married or cohabiting, and had higher average income. They also reported higher procedural and interactional justice and self-rated health the year before retirement. Those who retired early were more likely to be married/cohabiting and reported the lowest levels of procedural and interactional justice the year before retirement and the poorest self-rated health.

### 3.2. Results of Latent Growth Curve Analyses

In the first step of analysis, we aimed to estimate trajectories of procedural justice, interactional justice and self-rated health prior to retirement. The model fit indices, as well as the parameter estimates (intercept and slope) are provided in Table 3.

#### 3.2.1. Fit and Selection of the LGCM Models

The first model (Model 0) assessed a fixed intercept and indicated poor overall fit for all procedural and interactional justice, as well as for self-rated health based on the previously mentioned goodness of fit criteria (Table 3). The second model (Model 1) examined a random intercept. The fit of the model indicated a good overall fit for self-rated health (3 or 4 waves) but a rather mixed picture for procedural and interactional justice (SRMR for procedural justice: 0.085; RMSEA for interactional justice: 0.055). The next model (Model 2) examined random intercept and linear fixed slope. The values of the fit indices were below the theoretical cut-off values for all outcomes, indicating a good fit. The final model (Model 3) assessed both random intercept and random slope and indicated excellent overall fit for all the outcomes. For procedural and interactional justice, the significant difference in the chi-square tests suggested the superiority of the random intercept and slope model (Model 3) over Model 2 and Model 1.

For self-rated health over four waves, the chi2-difference tests indicated that while Model 3 fitted better than Model 2, Model 2 did not fit better than Model 1. In addition, the CFI and RMSEA values for Model 3 indicated a perfect fit, but the SBIC value was lowest for Model 1. Thus, taking into consideration all these pieces of information, we concluded that for self-rated health over four waves, Model 1 was superior. For self-rated health over three waves, a similar picture emerged. The SBIC value was lowest for Model 1 (the random intercept model), and the fit indices for Model 1 indicated excellent fit. We therefore concluded Model 1 to be superior even for self-rated health over three waves.

#### 3.2.2. Development of Justice and Health prior to Retirement

For procedural justice, we found a significant positive slope, which means that there was a linear decrease in perceptions of procedural justice in the years prior to retirement (as time was set to run backwards from the point of retirement). For interactional justice, we found a significant negative slope, which signals an opposite development of linear increase in perceived interactional justice during the working time prior to retirement. For self-rated health, the slope was not significant.

The models furthermore indicate that there was significant variance around the mean for both the slopes and the intercepts (except for the variance for the slope of self-rated health across three waves). This means that the intercept level of self-rated health over four waves varied considerably prior to retirement, but as indicated by the slope mean of 0.00, self-rated health did not significantly decline or increase during the years prior to retirement. For self-rated health across three waves, the non-significant slope and slope variance indicate that there was no significant increase or decrease during the years prior to retirement.

### 3.3. Results of Multinomial Logistic Regressions

In the second step of our analysis, we aim to examine how the LGCM of procedural and interactional justice over time may predict retirement groups.

#### 3.3.1. Comparison Late to Early Retirement Group

Results of the multinomial logistic regressions comparing late to early retirees are displayed in Table 4. For procedural justice, intercept levels among late as compared to early retirees were statistically significantly higher in the crude model (Model 1) but not when controlling for self-rated health and demographic characteristics (Model 2 and 3). Further, the slope of procedural justice was significant in the models controlling for self-rated health and demographic characteristics (Model 2 and Model 3), such that early retirees reported a steeper decline in procedural justice than late retirees. For interactional justice, late retirees reported significantly higher intercept levels of interactional justice in the years prior to retirement compared to early retirees (all models), thus, interactional justice levels remained significant when including self-rated health and demographic characteristics (Model 3). The slope of interactional justice was not statistically significant in any model. Late retirees reported significantly higher intercept levels of self-rated health than early retirees in all models. Thus, later compared to early retirees had higher intercept levels of interactional justice and self-rated health, and their procedural justice perceptions declined less steeply.

#### 3.3.2. Comparison Late to the Normative Retirement Group

The results of multinomial logistic regressions comparing late to the normative retirement group are displayed in Table 5. Late retirees had significantly higher intercept levels of interactional justice prior to retirement, and this finding remained the same when self-rated health and demographic characteristics were added in the model (Model 3). No significant differences between the two groups were found for the intercept levels of procedural justice or the slope of both justice dimensions. Moreover, there were no differences in intercept levels of self-rated health between late retirees compared to the normative retirement group.

#### 3.3.3. Comparison Early to the Normative Retirement Group

Compared to the normative retirement group (see Table 6), early retirees showed no significant differences in intercept and slope of procedural or interactional justice. Early retirees reported significantly lower intercept values of self-rated health compared to the normative retirement group. Table 4. ORs for late retirement as compared to early retirement (reference) for procedural justice and interactional justice.

## 4. Discussion

This study investigated trajectories of procedural and interactional justice as predictors of three groups of retirees considering self-rated health and several important demographic characteristics. Overall, our findings suggest that trajectories in organizational justice perceptions and self-rated health prior to retirement differ, to some extent, between early, normative and late retirees. The main findings in the comparison between late compared to early retirees are that late retirees had higher average levels of interactional justice, a lower decrease in procedural justice in the years prior to retirement and higher self-rated health prior to retirement. Late retirees also had higher average levels of interactional justice compared to the normative retirement group. Another main finding is that early retirees had lower self-rated health than those retiring at the normative retirement age. Retirement groups differed also according to demographic characteristics, particularly marital/cohabiting status and income. Thus, overall, the results point towards the fact that health may be a more important predictor of early retirement, while organizational justice perceptions may be more important for the decision to continue working past the normative retirement age.

There is a scarcity of studies predicting actual retirement behavior. Previous research has mainly focused on retirement intentions, which are different from actual retirement behavior [10]. Prospective studies from Denmark and Finland showed that workers with lower justice perceptions were more likely to retire early and receive disability pension [22,23,24]. We are not aware of previous studies investigating justice perceptions as predictor of actual late retirement. The novelty of the present study is that we examined the role of both levels and developments in procedural and interactional justice prior to retirement, and by further differentiating early, normative and also late retirement groups. We found that procedural justice perceptions decreased already several years before retirement among both those who retire early and those who retire at the normative retirement age. Results from the multinomial regression analyses indicate that this decrease is important, as the decrease in procedural justice had an association to retirement decision above and beyond self-rated health. Although self-rated health did not change much in the years prior to retirement for any of the three retirement groups, clear differences in the intercept levels of self-rated health between the retirees were revealed. While it is well-known in the literature that health is one of the major predictors of retirement decisions [8,32], our findings highlight that organizational justice perceptions, as indicator of the psychosocial work environment, can add to the decision to retire late.

Interestingly, the different justice dimensions showed slightly different developments over time in the three retirement groups. Interactional justice developed positively over time whereas procedural justice developed negatively over time. Unfortunately, the existing literature investigating the impact of organizational justice on retirement behavior is very limited [10], and the few existing studies often combined different subscales of organizational justice [22,23,26]. It may indeed be the case that interactional justice is more related to the relationship with the supervisor, which provides employees with clues about their standing within the working group. Interactional justice may, therefore, provide stronger information about self-evaluations than procedural justice, which, may, in turn, be more relevant to individuals’ retirement decisions. Another explanation of the differences in trajectories of procedural and interactional justice may relate to the ideas that different ages place different preferences to social contact, such that older ages place more importance to relational aspects and younger individuals more emphasis on instrumental aspects [44]. It is generally argued that justice perceptions fulfill psychological needs, and here procedural justice is likely to fulfill the need for control (instrumental need) whereas interactional justice fulfills the need of wanting to feel valued and respected (relational needs) [45]. However, the literature on how age shapes justice perceptions is limited. One study from the Netherlands showed that older workers were less likely to change their employer when they perceived high procedural justice and high trust to their supervisor [46]. The authors argued that trust in the leader is essential for older workers when deciding whether to stay or leave their organizations. While turnover is different from retirement, it is both a withdrawal behavior. Further research is needed in how different ages place relevance on different justice aspects, perceive different justice aspects and react to these justice perceptions.

Different pathways have been suggested to connect psychosocial work factors with retirement decisions [5]. One possible pathway is that high levels of job demands can, by exhausting mental and physical capacities, trigger work overload and subsequent poor health [5], which results in earlier retirement. This idea gets some support from our results. In our study, early retirees reported worse health than the other two groups. In this, our findings are in contrast to those reported in a systematic review, in which it was concluded that self-reported health only plays a marginal role for early retirement when not granted on health grounds [30]. However, other studies have shown that low self-rated health is a risk factor for early retirement and retirement intentions [47,48]. Such found Heponiemi, in that those with poorer self-rated health had higher odds to intend to retire early [26]. In addition, they also found that organizational injustice strengthened the associations of poor health and sickness absences with retirement intentions. Here, we did not investigate if the effect of self-rated health changed under different work environments, but found that the importance of justice perceptions on retirement decision was independent of self-rated health. The relationship between organizational justice and health is complex, with causality likely going both ways [49], such that low organizational justice perceptions can facilitate more ill-health over time, but that also lower health is associated with lower organizational justice perceptions. More longitudinal studies with a focus on actual retiring behavior (and not just intentions), that also differentiate between early and late retirement and look at the development of work environment factors over time, are needed.

Our results indicate also that certain demographic groups are more or less likely to retire late. Being not married or cohabiting and having a higher income were found to positively associate with *late* retirement. This is in line with previous research that have found that that married or cohabiting individuals tend to retire earlier [50]. Interestingly, sex did not differ between retirees in the regression analyses. This mimics the picture in the literature on retirement, where results of sex on retirement are mixed. More studies in other countries and different labor market contexts are needed to further elucidate on the impact of sex on retirement. Income had an interesting role, such that income was higher both among early and late retirees, as compared those retiring at the normative age. Some previous studies found that greater wealth predicts early retirement whereas others found that higher wealth can lead to delaying retirement, as well-paid white collar jobs may be less physical demanding and more stimulating [8]. However, our measure of income was based on income from employment and employment-related benefits solely, and did not take other forms of income into account.

Our study is, to our knowledge, the first to investigate organizational justice trajectories over time in relation to early, normative and late retirement. Strengths include that the data come from a diverse sample of occupations and professions, in contrast to the existing studies, which are mainly situated in the hospital and civil servant sector [22,23,24]. A further advantage is the longitudinal nature of the dataset with several points of measurements. A further strength is the consideration of actual retirement behavior (instead of intentions), and here the differentiation between different types of retirement behavior. Moreover, we investigated changes in two indicators of justice perceptions; procedural and interactional justice, as well as self-rated health as antecedents of said behavior. However, our study also has some limitations that need to be mentioned. First, our study sample was drawn from a cohort study originally focusing on the working population. Thus, while the sample is thus approximately representative of the working population in Sweden, our retirement groups were rather small in comparison to other studies. In addition, because the focus was on the working population at baseline and because we excluded participants with disability pension, the results may be biased in terms of a healthy worker effect. This is a common issue in studies on retirement, as individuals with poor health do not remain in work [8]. Moreover, retiring is a highly complex decision, and the process is impacted by many different factors [48], many of which could not be considered. Furthermore, similar studies should attempt to replicate the findings in other countries, labor market contexts and pension systems. The current literature on actual retirement behavior is surprisingly scarce, despite the societal relevance of understanding factors that contribute to retirement decisions of older workers. In terms of organizational justice facets, there is currently no study incorporating distributive justice and retirement behavior. As this study points out different justice facets show slightly different results, which supports looking at all justice facets as well other work environment factors in the future. In this study, we focused on procedural justice and interactional justice as well as self-rated health (and several demographic characteristics). Other studies may incorporate other work environment factors in addition to organizational justice facets and other health indicators to provide a fuller picture of predictors of retirement behavior. As our study indicated relevant differences between retirement decisions, we would urge researchers to also categorize retirement behavior into different categories, such as early, normative and late retirement.

Another limitation is the definition of retirement. Retirement is commonly operationalized in terms of age relative to the age at which one is eligible for pension [8]. Our allocation of group membership, however, is complicated by the fact that data collection was spaced out to two years and age registered at the end of the year, which makes an exact match of retirement at or around the 65th birthday complicated. As a result, we used somewhat broader categories of classifying everyone as normative who retired after the age of 64 and before the age of 66, and early/late retirement before and after that, respectively. Another option for future research studies would be to take into consideration data on which type of pension participants receive. With this, this study only considered those who indicated that they retired fully, thereby excluding any forms of bridge employment. However, there is some evidence that the decision to retire fully or engage in bridge employment is predicted more by non-work reasons than work-reasons [51]. Furthermore, this study is limited in that we did not measure if retirement was voluntary or forced (e.g., due to reorganization). As the group of late retirees included those who were still working past their 66th birthday (so have not technically retired yet), we re-ran the analyses without these individuals as a kind of sensitivity analyses; results revealed surprisingly similar results despite the loss of power.

## 5. Conclusions

Despite these limitations, this study contributes to the scarce literature on the impact of psychosocial work environment factors as predictors of retirement decisions using several points of measurement. Our findings provide some support that the level and the change in organizational justice perceptions over time can affect individuals’ retirement decisions, especially the decision to continue working after the normative retirement age and under the condition that self-rated health is fairly good. Given that many countries are desperate to increase retirement age and employees need to work likely longer in the future [9], these present findings suggest that justice perceptions at work may be one contributing factor to help achieve higher rates of late retirement. In practice, organizations need to actively work on creating fair workplaces by incorporating aspects of justice in key practices, like recruitment procedures, performance appraisal and reward systems, and managerial trainings. Our study shows that more attention needs to be directed to organizational justice with respect to interactions and procedures if older workers are asked to work longer. If policy-makers increase the retirement age, it is important to support organizations in creating a healthy work environment even for older workers, as well as counteracting the still widespread negative stereotypes of older workers. This may help maintain high levels of justice perceptions of older workers, and since justice has been found to be important for health, even keep the older worker healthier. Our study shows that both health and justice developments prior to retiring associate with the timing of retirement, but for the research community an important future avenue is to study in more detail how justice perceptions and health interact, and whether they are causal to decisions for retiring early, at the normative age or later.

## Figures and Tables

**Table 1:** Justice items.

**Procedural Justice**
1 Decision are taken on the basis of correct information
2 Bad decisions can be revoked or changed
3 All sides affected by the decision are represented
4 Decisions taken are consistent (the same rules apply to everyone)
5 Everyone is entitled to give their opinion in matters of immediate personal concern
6 Feedback is provided regarding the consequences of decisions and people are informed accordingly
7 It is possible to obtain a more detailed account of the information that underlies decisions, if needed
**Interactional Justice**
1 I receive praise from my boss if I have done something good
2 My boss shows that he/she cares how things are for me and how I feel.
3 My boss encourages my participation in the scheduling of my work.
4 My boss takes the time to become involved in his/her employees’ professional development.
5 My boss gives me the information I need.
6 I have a clear picture of what my boss expects of me.
7 My boss explains goals and sub-goals for our work so that I understand what they mean for my particular part of the work

**Table 2:** Description of retirement groups.

Variable	Normative N = 1231	Early N = 952	Late N = 817	*p* Value
Women % (n)	55.5 (683)	56.2 (535)	47.0 (384)	<0.0001
Age at retirement mean (SD)	65.23 (0.42)	62.82 (1.48)	67.73 (1.32)	<0.0001
White-collar worker % (n)	66.5 (794)	71.7 (669)	74.1 (585)	<0.001
Married/cohabiting % (n)	78.3 (945)	86.3 (816)	72.2 (582)	<0.0001
Income (two waves prior retirement) mean (SD)	372.80 (164.90)	412.76 (464.94)	475.93 (231.58)	<0.0001
Procedural justice (one wave before retirement or current) mean (SD)	3.36 (0.94)	3.27 (0.93)	3.50 (0.92)	<0.0001
Interactional justice (one wave before retirement or current) mean (SD)	3.13 (0.64)	3.10 (0.67)	3.26 (0.62)	<0.0001
Self-rated health (one wave before retirement or current) mean (SD)	4.04 (0.75)	3.96 (0.79)	4.13 (0.75)	<0.0001

Notes. For sex, socioeconomic status, civil status, chi-square tests were conducted. For age, income, procedural and interactional justice and self-rated health, ANOVAs were conducted.

**Table 3:** Model comparisons for creating intercepts and slopes including means and variances for intercepts and slopes.

Models	SBIC	χ^2^ (df)	Change in χ^2^	CFI	RMSEA	SRMR	I Mean	I Variance	S Mean	S Variance
Procedural justice 4 waves										
Model 0: fixed intercept	18,478.03	1831.27 (9)	-	0.000	0.265	0.360	3.41 ***			
Model 1: random intercept	16,699.76	48.20 (8)	-	0.978	0.042	0.085	3.40 ***	0.48 ***		
Model 2: random intercept, fixed slope	16,684.39	28.05 (7)	20.15 ***	0.988	0.032	0.066	3.37 ***	0.48 ***	0.04 ***	
Model 3: random intercept, random slope	16,683.72	17.80 (5)	10.25 ***	0.993	0.030	0.039	3.37 ***	0.53 ***	0.04 ***	0.02 **
Self-rated health 4 waves										
Model 0: fixed intercept	17,116.37	2304.08 (9)	-	0.001	0.292	0.386	4.04 ***			
Model 1: random intercept	14,829.43	12.31 (8)	-	0.998	0.013	0.051	4.04 ***	0.36 ***		
Model 2: random intercept, fixed slope	14,834.01	12.07 (7)	0.24 ns	0.998	0.016	0.049	4.04 ***	0.36 ***	0.00	
Model 3: random intercept, random slope	14,834.37	2.78 (5)	9.29 *	1.000	0.000	0.018	4.04 ***	0.39 ***	0.00	0.01 **
Interactional justice 3 waves										
Model 0: fixed intercept	8767.10	951.74 (5)	-	0.000	0.285	0.334	3.16 ***			
Model 1: random intercept	7852.06	32.13 (4)	-	0.970	0.055	0.044	3.16 ***	0.24 ***		
Model 2: random intercept, fixed slope	7847.93	23.42 (3)	8.70 ***	0.978	0.054	0.036	3.17 ***	0.24 ***	−0.03 **	
Model 3: random intercept, random slope	7844.96	11.29 (1)	12.13 ***	0.989	0.066	0.027	3.17 ***	0.29 ***	−0.03 **	0.04 ***
Self-rated health 3 waves										
Model 0: fixed intercept	15,003.77	1791.18 (5)	-	0.000	0.346	0.351	4.04 ***			
Model 1: random intercept	13,222.46	5.04 (4)	-	0.999	0.009	0.041	4.04 ***	0.37 ***		
Model 2: random intercept, fixed slope	13,226.92	4.68 (3)	0.36 ns	0.999	0.014	0.039	4.04 ***	0.37 ***	0.01	
Model 3: random intercept, random slope	13,232.63	0.74 (1)	3.94 *	1.000	0.000	0.004	4.04 ***	0.40 ***	0.01	0.02

Notes. *** *p* < 0.001; ** *p* < 0.01, * *p* < 0.05. Change in χ^2^ Model 2 is compared to Model 1 and Model 3 is compared to Model 2.

**Table 4:** ORs for late retirement as compared to early retirement (reference) for procedural justice and interactional justice.

	Procedural Justice						Interactional Justice					
Variables	Model 1 N = 2880		Model 2 N = 2873		Model 3 N = 2760		Model 1 N = 2334		Model 2 N = 2332		Model 3 N = 2235	
	OR	95% CI	OR	95% CI	OR	95% CI	OR	95% CI	OR	95% CI	OR	95% CI
Intercept J	**1.04**	**1.01–1.07**	1.03	1.00–1.06	1.03	0.99–1.06	**1.84**	**1.41–2.39**	**1.67**	**1.28–2.18**	**1.67**	**1.27–2.21**
Slope J	0.68	0.46–1.01	**0.67**	**0.45–0.99**	**0.63**	**0.42–0.94**	1.17	0.31–4.49	1.13	0.29–4.36	0.98	0.24–3.95
Intercept SRH			**1.60**	**1.32–1.93**	**1.46**	**1.19–1.78**			**1.57**	**1.28–1.92**	**1.46**	**1.18–1.81**
Gender (male)					1.10	0.89–1.37					1.20	0.94–1.53
Married (not-married)					**2.83**	**2.18–3.66**					**2.58**	**1.95–3.41**
SES (lower)					1.24	0.97–1.60					1.18	0.90–1.55
Ln (Income)					**2.97**	**2.21–3.98**					**2.50**	**1.80–3.48**
−2 Log Likelihood	4581.36 ***		5256.67 ***		5739.76 ***		2720.30 ***		4180.63 ***		4703.86 ***	
Cox and Snell Pseudo R^2^	0.009		0.017		0.087			0.012		0.021		0.079

Notes. *** *p* < 0.001. Numbers in bold font are statistically significant with a 95% confidence interval. J = justice perception aspect; SRH = self-rated health; SES = socioeconomic status. Reference group is early retirement group.

**Table 5:** ORs for late retirement as compared to normative retirement (reference group) for procedural justice and interactional justice.

	Procedural Justice						Interactional Justice					
Variables	Model 1 N = 2880		Model 2 N = 2873		Model 3 N = 2760		Model 1 N = 2334		Model 2 N = 2332		Model 3 N = 2235	
	OR	95% CI	OR	95% CI	OR	95% CI	OR	95% CI	OR	95% CI	OR	95% CI
Intercept J	1.02	1.00–1.05	1.02	0.99–1.05	1.02	0.99–1.04	**1.65**	**1.28–2.11**	**1.55**	**1.21–2.00**	**1.44**	**1.10–1.88**
Slope J	0.83	0.57–1.20	0.82	0.56–1.18	0.78	0.53–1.15	1.34	0.37–4.80	1.30	0.36–4.66	1.12	0.30–4.19
Intercept SRH			**1.32**	**1.10–1.58**	1.12	0.92–1.36			**1.34**	**1.10–1.62**	1.18	0.96–1.45
Gender (male)					1.00	0.81–1.23					1.06	0.84–1.33
Married (not-married)					**1.51**	**1.21–1.89**					**1.52**	**1.19–1.93**
SES (lower)					1.10	0.87–1.40					1.07	0.83–1.37
Ln (Income)					**4.11**	**3.10–5.47**					**3.42**	**2.48–4.71**
−2 Log Likelihood	4581.36 ***		5256.67 ***		5739.76 ***		2720.30 ***		4180.63 ***		4703.86 ***	
Cox and Snell Pseudo R^2^	0.009		0.017		0.087		0.012		0.021		0.079	

Notes. *** *p* < 0.001. Numbers in bold font are statistically significant with a 95% confidence interval. J = justice perception aspect; SRH = self-rated health; SES = socioeconomic status. Reference group is normative retirement group.

**Table 6:** ORs for early retirement as compared to normative retirement (reference group) for procedural justice and interactional justice.

	Procedural Justice						Interactional Justice					
Variables	Model 1 N = 2880		Model 2 N = 2873		Model 3 N = 2760		Model 1 N = 2334		Model 2 N = 2332		Model 3 N = 2235	
	OR	95% CI	OR	95% CI	OR	95% CI	OR	95% CI	OR	95% CI	OR	95% CI
Intercept J	0.99	0.96–1.01	0.99	0.97–1.02	0.99	0.96–1.01	0.90	0.70–1.14	0.93	0.73–1.19	0.86	0.67–1.11
Slope J	1.21	0.85–1.73	1.22	0.86–1.74	1.25	0.86–1.80	1.14	0.33–3.98	1.15	0.33–4.01	1.14	0.31–4.20
Intercept SRH			**0.82**	**0.70–0.97**	**0.77**	**0.65–0.91**			0.85	0.71–1.03	**0.82**	**0.67–0.98**
Gender (male)					0.91	0.74–1.10					0.88	0.70–1.10
Married (not-married)					**0.54**	**0.42–0.68**					**0.59**	**0.45–0.77**
SES (lower)					0.89	0.72–1.10					0.90	0.71–1.15
Ln (Income)					**1.39**	**1.06–1.81**					1.37	0.99–1.89
−2 Log Likelihood	4581.36 ***		5256.67 ***		5739.76 ***		2720.30 ***		4180.63 ***		4703.86 ***	
Cox and Snell Pseudo R^2^	0.009		0.017		0.087		0.012			0.021	0.079	

Notes. *** *p* < 0.001. Numbers in bold font are statistically significant with a 95% confidence interval. J = justice perception aspect; SRH = self-rated health; SES = socioeconomic status. Reference group is normative retirement group.

## Data Availability

The datasets generated and/or analyzed during the current study are not publicly available due legal restrictions but are available from the corresponding author on reasonable request. Data is available upon reasonable request.

## References

[1] Eurostat Projected Old-Age Dependency Ratio. https://ec.europa.eu/eurostat/web/products-eurostat-news/-/ddn-20200713-1#:~:text=The%20EU’s%20old%2Dage%20dependency,person%20aged%2065%20and%20over.

[2] OECD (2019). Pensions at a Glance 2019: OECD and G20 Indicators.

[3] Anxo D., Ericson T., Herbert A., Rönnmar M. (2017). To Stay or Not to Stay. That Is the Question: Beyond Retirement: Stayers on the Labour Market.

[4] Statens Offentliga Utredningar (2020). Äldre har Aldrig Varit Yngre—Allt Fler Kan Och Vill Arbeta Längre. Betänkande av Delegationen för Senior Arbetskraft.

[5] Carr E., Hagger-Johnson G., Head J., Shelton N., Stafford M., Stansfeld S., Zaninotto P. (2016). Working conditions as predictors of retirement intentions and exit from paid employment: A 10-year follow-up of the English Longitudinal Study of Ageing. Eur. J. Ageing.

[6] Stansfeld S.A., Carr E., Smuk M., Clark C., Murray E., Shelton N., Head J. (2018). Mid-life psychosocial work environment as a predictor of work exit by age 50. PLoS ONE.

[7] Feldman D.C., Beehr T.A. (2011). A three-phase model of retirement decision making. Am. Psychol..

[8] Fisher G.G., Chaffee D.S., Sonnega A. (2016). Retirement Timing: A Review and Recommendations for Future Research. Work Aging Retire..

[9] Wang M., Shi J. (2014). Psychological Research on Retirement. Annu. Rev. Psychol..

[10] Browne P., Carr E., Fleischmann M., Xue B., Stansfeld S.A. (2019). The relationship between workplace psychosocial environment and retirement intentions and actual retirement: A systematic review. Eur. J. Ageing.

[11] Rupp D.E., Shapiro D., Folger R., Skarlicki D., Shao R. (2017). A Critical Analysis of the Conceptualization and Measurement of ‘Organizational Justice’: Is it Time for Reassessment?. Acad. Manag. Ann..

[12] Colquitt J.A., Scott B.A., Rodell J.B., Long D.M., Zapata C.P., Conlon D.E., Wesson M.J. (2013). Justice at the millennium, a decade later: A meta-analytic test of social exchange and affect-based perspectives. J. Appl. Psychol..

[13] Colquitt J.A. (2001). On the dimensionality of organizational justice: A construct validation of a measure. J. Appl. Psychol..

[14] Scott B.A., Colquitt J.A., Paddock L. (2009). An actor-focused model of justice rule adherence and violation: The role of managerial motives and discretion. J. Appl. Psychol..

[15] Robbins J.M., Ford M.T., Tetrick L.E. (2012). Perceived unfairness and employee health: A meta-analytic integration. J. Appl. Psychol..

[16] Ndjaboué R., Brisson C., Vézina M. (2012). Organisational justice and mental health: A systematic review of prospective studies. Occup. Environ. Med..

[17] Elovainio M., Kivimäki M., Vahtera J. (2002). Organizational justice: Evidence of a new psychosocial predictor of health. Am. J. Public Health.

[18] Tyler T.R., Blader S.L. (2003). The group engagement model: Procedural justice, social identity, and cooperative behavior. Personal. Soc. Psychol. Rev..

[19] Blau P.M. (1964). Exchange and Power in Social Life.

[20] Leineweber C., Peristera P., Bernhard-Oettel C., Eib C. (2020). Is interpersonal justice related to group and organizational turnover? Results from a Swedish panel study. Soc. Sci. Med..

[21] Colquitt J.A., Conlon D.E., Wesson M.J., Porter C.O.L.H., Ng K.Y. (2001). Justice at the millenium: A meta-analytic review of 25 years of organizational justice research. J. Appl. Psychol..

[22] Breinegaard N.P., Jensen J.H.M., Bonde J.P.P. (2017). Organizational change, psychosocial work environment, and non-disability early retirement: A prospective study among senior public employees. Scand. J. Work Environ. Health.

[23] Thorsen S.V., Jensen P.H., Bjørner J.B. (2016). Psychosocial work environment and retirement age: A prospective study of 1876 senior employees. Int. Arch. Occup. Environ. Health.

[24] Juvani A., Oksanen T., Virtanen M., Elovainio M., Salo P., Pentti J., Kivimäki M., Vahtera J. (2016). Organizational justice and disability pension from all-causes, depression and musculoskeletal diseases: A Finnish cohort study of public sector employees. Scand. J. Work Environ. Health.

[25] Harkonmäki K., Rahkonen O., Martikainen P., Silventoinen K., Lahelma E. (2006). Associations of SF-36 Mental Health Functioning and Work and Family Related Factors with Intentions to Retire Early among Employees. Occup. Environ. Med..

[26] Heponiemi T., Kouvonen A., Vänskä J., Halila H., Sinervo T., Kivimäki M., Elovainio M. (2008). Health, psychosocial factors and retirement intentions among Finnish physicians. Occup. Med..

[27] Sulander J., Sinervo T., Elovainio M., Heponiemi T., Helkama K., Aalto A.-M. (2016). Does Organizational Justice Modify the Association Between Job Involvement and Retirement Intentions of Nurses in Finland?. Res. Nurs. Health.

[28] Sutinen R., Kivimäki M., Elovainio M., Forma P. (2005). Associations between stress at work and attitudes towards retirement in hospital physicians. Work Stress.

[29] van den Berg T., Schuring M., Avendano M., Mackenbach J., Burdorf A. (2010). The impact of ill health on exit from paid employment in Europe among older workers. Occup. Environ. Med..

[30] van Rijn R.M., Robroek S.J., Brouwer S., Burdorf A. (2014). Influence of poor health on exit from paid employment: A systematic review. Occup. Environ. Med..

[31] Jokela M., Ferrie J.E., Gimeno D., Chandola T., Shipley M.J., Head J., Vahtera J., Westerlund H., Marmot M.G., Kivimaki M. (2010). From Midlife to Early Old Age: Health Trajectories Associated with Retirement. Epidemiology.

[32] Westerlund H., Kivimäki M., Singh-Manoux A., Melchior M., Ferrie J.E., Pentti J., Jokela M., Leineweber C., Goldberg M., Zins M. (2009). Self-rated health before and after retirement in France (GAZEL): A cohort study. Lancet.

[33] Stenholm S., Virtanen M., Pentti J., Oksanen T., Kivimäki M., Vahtera J. (2020). Trajectories of self-rated health before and after retirement: Evidence from two cohort studies. Occup. Environ. Med..

[34] Magnusson Hanson L.L., Leineweber C., Persson V., Hyde M., Theorell T., Westerlund H. (2018). Cohort Profile: The Swedish Longitudinal Occupational Survey of Health (SLOSH). Int. J. Epidemiol..

[35] Moorman R.H. (1991). Relationship between organizational justice and organizational citizenship behaviors: Do fairness perceptions influence employee citizenship?. J. Appl. Psychol..

[36] Setterlind S., Larsson G. (1995). The stress profile: A psychosocial approach to measuring stress. Stress Med..

[37] Idler E.L., Benyamini Y. (1997). Self-rated health and mortality: A review of twenty-seven community studies. J. Health Soc. Behav..

[38] Burant C.J. (2016). Latent Growth Curve Models: Tracking Changes Over Time. Int. J. Aging Hum. Dev..

[39] Bollen K.A., Curran P.J. (2006). Latent Curve Models: A Structural Equation Perspective.

[40] Muthén L.K., Muthén B.O. Mplus User’s Guide.

[41] Bentler P.M. (1990). Comparative fit indexes in structural models. Psychol. Bull..

[42] Hu L.-t., Bentler P.M. (1999). Cutoff Criteria for Fit Indexes in Covariance Structure Analysis: Conventional Criteria versus New Alternatives. Struct. Equ. Modeling.

[43] Tabachnick B.G., Fidell L.S. (2007). Using Multivariate Statistics.

[44] Brienza J.P., Bobocel D.R. (2017). Employee Age Alters the Effects of Justice on Emotional Exhaustion and Organizational Deviance. Front. Psychol..

[45] Cropanzano R., Byrne Z.S., Bobocel D.R., Rupp D.E. (2001). Moral virtues, fairness heuristics, social entities, and other denizens of organizational justice. J. Vocat. Behav..

[46] Bal P.M., de Lange A.H., Ybema J.F., Jansen P.G.W., van der Velde M.E.G. (2011). Age and trust as moderators in the relation between procedural justice and turnover: A large-scale longitudinal study. Appl. Psychol. Int. Rev..

[47] Friis K., Ekholm O., Hundrup Y.A., Obel E.B., Grønbæk M. (2007). Influence of health, lifestyle, working conditions, and sociodemography on early retirement among nurses: The Danish Nurse Cohort Study. Scand. J. Public Health.

[48] de Preter H., van Looy D., Mortelmans D. (2013). Individual and institutional push and pull factors as predictors of retirement timing in Europe: A multilevel analysis. J. Aging Stud..

[49] Lang J., Bliese P.D., Lang J.W.B., Adler A.B. (2011). Work gets unfair for the depressed: Cross-lagged relations between organizational justice perceptions and depressive symptoms. J. Appl. Psychol..

[50] Madero-Cabib I., Kaeser L. (2016). How voluntary is the active ageing life? A life-course study on the determinants of extending careers. Eur. J. Ageing.

[51] Bennett M.M., Beehr T.A., Lepisto L.R. (2016). A longitudinal study of work after retirement: Examining predictors of bridge employment, continued career employment, and retirement. Int. J. Aging Hum. Dev..

